# Glances at the Hospitals

**Published:** 1905-06-10

**Authors:** 


### GLANCES AT THE HOSPITALS.

### THE KEIGHLEY HOSPITAL.

The total receipts amounted to £1,787 and the total expenditure to £1,733, a statement which shows that the expenditure has been kept well within the income and therefore it may fairly be assumed that the management is good and that subscriptions and donations may be safely entrusted to the board of managers for careful outlay. On the first of January the hospital contained 23 patients, and 349 were admitted during the year, making a total of 372 under treatment. The average daily number of beds occupied was 32, and the average duration of residence was 33 days. The cost for each patient for treatment, nursing, and maintenance was 2s. 9d. a day. The hospital is being extended, and the committee appeal for subscriptions to enable them to complete the additions. £2,586 have been received, but over £1,500 of this was given by Messrs. J. Clough and Sons, Mr. and Mrs. S. Clough, and Miss Clough, and Mrs. Emmott gave £275. It is proposed to hold a bazaar this year in aid of this fund, and in the meantime it is to be hoped that the inhabitants of Keighley and neighbourhood will subscribe handsomely and follow the generous leads of the donors named above. The medical and surgical tables are unusually well drawn out for a small hospital. During the year there were 8 cases of ovarian tumour under treatment, and 5 of these were cured, 1 died, and 2 remained in the wards. The operation for appendicitis was performed 7 times, that of the radical cure of hernia 9 times, and that for strangulated hernia 5 times. It is a pity that the results of these operations are not given. A summary of the surgical cases shows that 241 were admitted, 195 cured or relieved, 7 not relieved, 7 discharged for other reasons, 14 died, and 18 remained in the hospital. On the medical side of the house 108 were admitted, 77 were cured, 3 not relieved, 3 were discharged at own request, 14 died, and 11 remained in the wards. The anæsthetic table tells us that ether was given 20 times, chloroform 269, ether and chloroform 6, ether and A.C.E. twice, chloroform followed by ether 4 times, chloroform followed by C.E. mixture twice, A.C.E. mixture once, cocaine 3 times, beta-eucain once, and ethyl chloride once. The total number of patients put under anæsthetics was 309, and no death followed. Private and district nurses can be supplied by the hospital. The latter are charged for at from 4d. to 1s. a visit according to the rental of the house visited.

